# A Schematic Colorimetric Assay for Sialic Acid Assay Based on PEG-Mediated Interparticle Crosslinking Aggregation of Gold Nanoparticles

**DOI:** 10.3390/bios13020164

**Published:** 2023-01-20

**Authors:** Shixing Tang, Lin Li, Rui Wang, Sagar Regmi, Xinyu Zhang, Guoqiang Yang, Jian Ju

**Affiliations:** 1 School of Ophthalmology & Optometry, Wenzhou Medical University, Wenzhou 325035, China; 2 Engineering Research Center of Clinical Functional Materials and Diagnosis & Treatment Devices of Zhejiang Province, Wenzhou Institute, University of Chinese Academy of Sciences, Wenzhou 325001, China; 3 Department of Epidemiology, School of Public Health, Southern Medical University, Guangzhou 510515, China; 4 Department of Pharmacology, School of Medicine, Case Western Reserve University, Cleveland, OH 44106, USA; 5 Key Laboratory of Photochemistry, Institute of Chemistry, University of Chinese Academy of Sciences, Chinese Academy of Sciences, Beijing 100190, China; 6 Oujiang Lab, Wenzhou 325001, China

**Keywords:** colorimetric assay, gold nanoparticles, 4-Mercaptophenyl boronic acid, polyethylene glycol 400, repulsive the interparticle crosslinking, sialic acid

## Abstract

Sialic acid (SA) is a well-known component of glycoproteins, which have applications in various functional processes on the cell’s surface. The colorimetric is a simpler and more convenient method for measuring SA due to its low-cost apparatus and visual signal changes. This work focused on the unpredictable interparticle crosslinking aggregation of the functionalized gold nanoparticles (AuNPs) in complex media. We proposed a balance of the Derjaguin–Landau–Verwey–Overbeek (DLVO)-type aggregation and molecule-based interaction method to solve this problem. Here, we report a novel colorimetric assay for the determination of SA using 4-mercaptophenyl boronic acid (4-MPBA) as an analyte’s recognition molecule, and negative charge PEG_400_ was used to repulsive the interparticle crosslinking. The proposed sensing platform shows a linear relationship between the ratio of the absorbance intensity (A_525_/A_660_) and concentration of SA from 0.05 to 8 mM (R^2^ = 0.997) and a detection limit of 48 μM was observed. The novel gold-based colorimetric sensor is easy to fabricate, reproducible in its test performance and has been successfully applied for the detection of SA in biological and healthcare product samples.

## 1. Introduction

N-Acetylneuraminic Acid (Neu5Ac or NANA), which contains nine carbon carbohydrates α-Ketoacid, is the most widely distributed sialic acid (SA) in nature, which is ubiquitous in vertebrates and mammals. The SA is an important component of glycoprotein that is densely displayed on the membrane of mammalian cells [1] that regulates the critical processes in receiving external information, cell adhesion, and antigenicity [2]. The research suggests that SA has a variety of physiological functions and plays a significant role in regulating human physiological and pathological processes [3,4], such as promoting brain development, transporting ions, stabilizing protein on the membrane and signal transduction, etc. [5]. Moreover, the expression level of SA provides important information and serves as a biomarker in tumor malignancy, diabetic symptoms, and other SA-associated disease diagnoses [6,7]. Currently, many efforts have been undertaken to monitor the free forms of SA in biological fluids, such as serum, to understand clinical diagnosis and screening of the disease [8]. In recent years, many studies found that a huge amount of SA may fall off the diseased cell’s surface and enter the blood, which increases the level of serum SA and is positively related to the severity of the disease [9,10]. Thus, it is very important to sensitivity-detect SA in the serum sample, which is helpful in disease screening and follow-up and in monitoring treatment [9]. In addition, the previous study indicated that exogenous SA via oral administration has helped to increase the repair of the cell tissue, promoting cell division and cell proliferation [11,12]. Interestingly, SA has also been reported to be effective in the treatment of Parkinson’s disease and Alzheimer’s disease [13]. More studies have revealed that SA as a dietary supplement can be used as a cognitive enhancer [14]. Premium bird’s nest has been regarded as rare healthcare food for a dietary supplement of SA. Therefore, assured conformance with healthcare requires an accurate and highly efficient approach for detecting trace levels of SA in the bird’s nest samples and dietary products.

Previously, some analytical methods were dedicated to the detection and evaluation of SA based on colorimetric [15,16], fluorescent [17], enzymatic method [18], electrochemical biosensor [19,20], enhanced Raman scattering [21], quartz crystal microbalance (QCM) [22] and chromatographic assays [23,24]. However, those methods have some limitations, such as overly expensive instrumentation, complicated analysis steps, time-consuming and high proficiency requirements for operators. Compared with the other methods, the colorimetric was simpler and more convenient to detect SA [15]. Furthermore, no special cost apparatus was needed, and the results can be observed by the naked eye [16].

Colloidal gold nanoparticles (AuNPs) are frequently used to design colorimetric sensor for their biofunctionalization, biostability and spectral properties [25]. Depending on the plasmon resonance of AuNPs, controlling the size and aggregation of the particles, the change of color of the colloidal solution can be observed, which provides a platform for colorimetric detection of a variety of analytes [26]. Based on the Derjaguin–Landua–Verwey–Overbeck (DLVO) theory, the stabilization of the AuNPs can be governed by the balance between the attractive van der Waals force and repulsive electrostatic double-layer force of the adjacent particles approaching each other [27].

However, the aggregation state of AuNPs can be initiated by several uncontrollable factors in the complex test system, such as the concentration change of the salt, the electrostatic disturbances from charged biomolecules, or nonspecific binding of the biological components with the unprotected active site, which potentially impair the performance of the measurement [28,29]. Therefore, more and more research focused on designing a non-DLVO-type aggregation system to precisely induce either aggregation or dispersion of the AuNPs is essential [28]. In this instance, the metal NPs’ surface-modified with various small molecules, polymers or polyelectrolytes (e.g., Au-thiol, Au-amine), can be used with the chemical coupling method [30,31]. In addition, metal NPs are also used to control the surface charge of the AuNPs—Bastami et al. first reported using the silver citrate and their oxygen functional groups to achieve the negative surface charge of the Au@Ag NPs [32]. In this case, the prevention of interparticle crosslinking aggregation caused by the molecule-based interaction in the absence of the target analyte has become a new challenge.

**Scheme 1:**
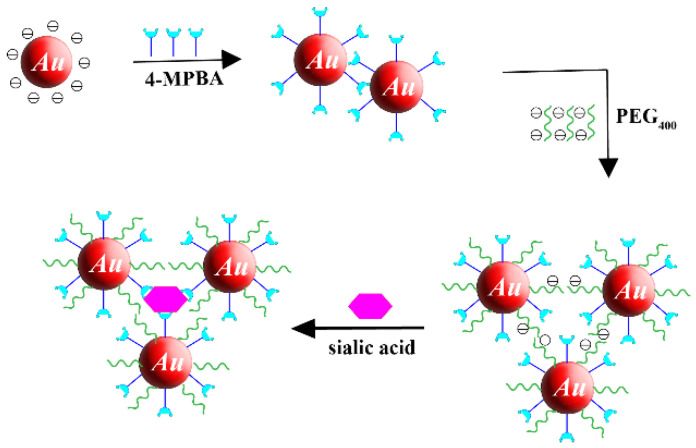
Schematic illustration of the synthetic strategy of 4-MPBA-AuNPs@PEG_400_ and the mechanism of the probe for SA detection.

In this work, we developed a novel strategy for the design of stability of AuNPs-based colorimetric sensing platform by the balance between DLVO-based and molecule-based interaction. As shown in Scheme 1, this kind of sensing for the detection of SA relies on the 4-Mercaptophenyl boronic acid (MPBA) modified on the AuNPs’ surface used as the recognition receptor. The principle of specific SA detection capitalizes on aryl-boronic compounds as a ligand for the affinity selection of SA. To regulate interparticle crosslinking aggregation of the functionalized AuNPs, a negative charge of polyethylene glycol 400 (PEG_400_) was filled on the unprotected gold surface to overcome the interparticle crosslinking. Our experimental results show that the measurement systems exhibited an enhanced test performance by the switches between the governing parameters of the two types of interactions. The proposed method provides a rapid, mild and green approach for the preparation of 4-MPBA-AuNPs@PEG_400_, and the stable measurement sensing systems exhibited high sensitivity for the detection of SA. They can be an outstanding colorimetric sensor for the selective recognition of SA among other glycan constituent saccharides. Interestingly, the biocompatible hydrophilic PEG400 has a strong hydration layer to prevent some biomolecule adsorption or bacterial adhesion to the functionalized AuNPs’ surface, which is advantageous for complex biological sample detection applications.

## 2. Materials and Methods

### 2.1. Chemical Reagents

The citric acid (CA), 4-mercaptophenyl boronic acid (4-MPBA), Ethanol and potassium hydroxide (KOH) were purchased from Aladdin Shanghai, China. Gold (III) chloride solution (HAuCl_4_) and tri-sodium citrate (C_6_H_5_O_7_Na_3_) were bought from Energy Chemical Shanghai, China. Polyethylene glycol PEG-400 and dimethyl sulfoxide were purchased from Macklin Shanghai, China. N-acetylneuraminic acid (Neu5Ac) was from Sigma-Aldrich (St. Louis, MO, USA). Nanopure water (18.3 MΩ cm) was used throughout the experiments.

### 2.2. Characterizations

The morphology, functionalization and size of the AuNPs were characterized by a high-resolution-transmission electron microscope (HRTEM, FEI Talos F200S, Hillsboro, OR, USA). The surface functional groups on AuNPs were obtained from a Raman spectrometer with 633 nm laser excitation (Renishaw, London, UK). The UV–Vis absorption spectrum was performed on a TU-1901 spectrometer (Beijing, China). The zeta potentials and dynamic light scattering (DLS) analysis were carried out by a Nano ZS ZEN3600 Zetasizer (Malvern, UK).

### 2.3. AuNPs Synthesis

The AuNPs were prepared by the adapted trisodium citrate reduction method [15]. Briefly, 2 mL of 1% HAuCl_4_ solution was added to 98 mL boiling water, followed by the rapid addition of 8 mL of 1% trisodium citrate solution. The color of the solution changed from colorless to wine-red, which indicated the Au^3+^ reduction to Au^0^. The solution was boiled for 10 min, the heater was turned off, and the solution was stirred vigorously for 15 min. Then, the reaction container was removed from the heat source and cooled to room temperature. The volume was fixed to 100 mL, and the solution was stored at 4 °C until use.

### 2.4. Preparation of 4-MPBA-AuNPs and 4-MPBA-AuNPs@PEG_400_

The AuNPs functionalized with 4-MPBA (4-MPBA-AuNPs) were prepared by incubation at room temperature [33]. In short, 5 mL of the pH of the AuNPs solution was adjusted to 11.0 with 0.5 M KOH solution, then 10 μL 4-MPBA (0.05 M) was added into the above AuNPs solution to form 4-MPBA-AuNPs. The mixed solution was incubated for 12 h in rotation to ensure the completion of the reaction. Then, 0.25 mL of 1% (mass fraction) PEG_400_ was added dropwise into the prepared 4-MPBA-AuNPs solution, and the mixture solution was incubated for another 30 min at room temperature. To remove the unconnected modifying agent, the 4-MPBA-AuNPs@PEG_400_ solution was centrifugation at 7500 rpm for 30 min at 4 °C. After removing the supernatant, the precipitated 4-MPBA-AuNPs@PEG_400_ was redissolved with 1.0 × 10^−11^ M KOH and stored in a clean glass bottle for subsequent use. The presence of the 4-MPBA-AuNPs and 4-MPBA-AuNPs@PEG_400_ was confirmed by the UV–Vis absorption spectrum and Raman spectrometer system.

### 2.5. Colorimetric Sensing Analysis of SA

All the colorimetric detection was conducted under normal indoor conditions (average temperature of 25 °C and relative humidity of 55%). Firstly, the 4-MPBA-AuNPs@PEG_400_ solution was redissolved to the 0.01 M blank buffer solution (citric acid and KOH buffer, pH 5.6). Secondly, 3 mL of the above solution was moved to a quartz cuvette after adding different volumes of a solution containing SA. The UV-Vis absorption values were recorded at the optimal experimental condition, and the detection response was the absorbance ratio of A_525_/A_660_.

### 2.6. Real Sample Analysis

The dried bird’s nests were purchased from the local pharmacy. A pestle and mortar ground the dry bird’s nest into a fine powder. Then, 0.1 g of powder was dissolved in 10 mL of 50% acetic acid solution, followed by hydrolysis in a water bath at 100 °C for 10 min. The neutralized solution was then filtered with 0.22 μM membrane and stored at 4 °C refrigerator.

The serum samples of mice were obtained from Zhejiang Experimental Animal Center. All the serum sample collection was performed in compliance with the guidelines of the Wenzhou Institute, University of Chinese Academy of Sciences (WIUCAS) Animal Care and Use Committee under the protocol WIUCS22051102. After mixing the serum sample with 5.56 µL of sulfuric acid (18 M) to 994.44 µL serum sample, the sample was first hydrolyzed to release SA from the glycoprotein, followed by digestion at 80 °C for 60 min, then neutralized with 200 µL of KOH (0.50 M) before analysis [34].

## 3. Results and Discussion

### 3.1. Characterization of the AuNPs

**Figure 1:**
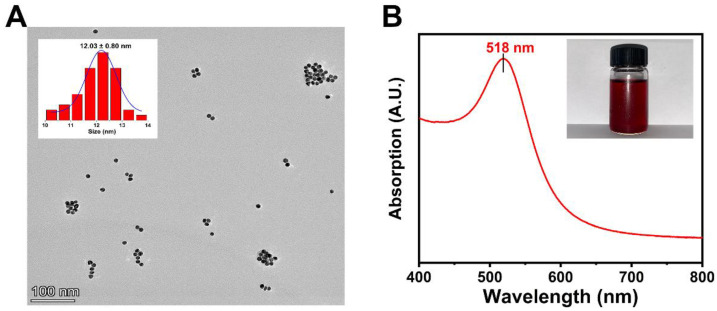
(**A**) The representative TEM micrograph of the AuNPs where the inset shows the histogram of the corresponding size distribution histogram. (**B**) The UV–Vis absorption spectra of AuNPs where the inset shows the representative image of AuNPs aqueous colloids.

The morphologies of the prepared AuNPs were characterized by HRTEM. The AuNPs exhibited a uniform particle size, and the average diameter of the AuNPs is around 12.03 ± 0.8 nm, as shown in Figure 1A. The UV–Vis absorption spectra displayed a single absorption peak of the colloidal AuNPs at around 518 nm is shown in Figure 1B, due to the surface plasmon resonance (SPR). This confirms the formation of uniform AuNPs according to the TEM image. The inset gave a visual representation of the brilliant wine-red color of the prepared colloidal AuNPs, resulting from the presence of uniform size and spherical AuNPs [35]. In this classical synthesis strategy, sodium citrate is used as a reductant and stabilizer; more importantly, the versatile citrate layer on the AuNPs’ surface provides more multi-functionalization possibilities.

### 3.2. Characterization of 4-MPBA-AuNPs and 4-MPBA-AuNPs@PEG_400_

The morphological and stability of the colloidal system 4-MPBA-AuNPs, and 4-MPBA-AuNPs@PEG_400_ was characterized by the TEM, Zeta potentials and UV–Vis absorption. The 4-MPBA-AuNPs were prepared by the modified optimized concentration of 4-MPBA (0.1 mol/L) on the surface of the AuNPs. The volume was 10 µL to obtain the optimized experiment for detecting SA. To avoid the interparticle crosslinking aggregation of the 4-MPBA-AuNPs, 250 µL of 1% PEG_400_ was filled on the AuNPs’ surface.

**Figure 2:**
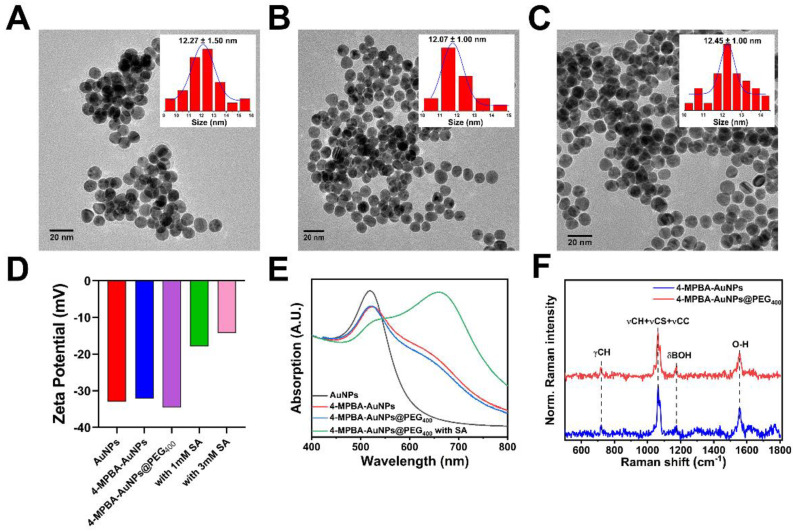
The representative TEM images of the (**A**) 4-MPBA-AuNPs, (**B**) 4-MPBA-AuNPs@PEG_400_, and (**C**) 4-MPBA-AuNPs@PEG_400_ with SA. (**D**) The zeta potential of AuNPs, 4-MPBA-AuNPs, 4-MPBA-AuNPs@PEG_400_, and 4-MPBA-AuNPs@PEG_400_ in the presence of 1 mM and 3 mM SA. (**E**) The UV–Vis absorption of the AuNPs, 4-MPBA-AuNPs, 4-MPBA-AuNPs@PEG_400_ and 4-MPBA-AuNPs@PEG_400_ mixed with 8 mM SA. (**F**) The Raman spectra of 4-MPBA-AuNPs and 4-MPBA-AuNPs@PEG_400_.

From illustrations of size distribution in Figure 2A–C, small molecules 4-MPBA, PEG_400_ and SA only form an oligolayer structure on the surface of AuNPs and have a negligible influence on the particle size. Nevertheless, the TEM images indicated an obvious crosslinking aggregation of the 4-MPBA-AuNPs compared with the citrate-stabilized AuNPs, as shown in Figure 2A. The PEG_400_ was introduced (4-MPBA-AuNPs@PEG_400_), and the distribution of agglomerated 4-MPBA-AuNPs tended to disperse as expected, as shown in Figure 2B. Interestingly, the 4-MPBA-AuNPs@PEG_400_ still retains the active sites for the connecting SA, as shown in Figure 2C. The TEM image gives reliable evidence that functionalized AuNPs display a denser aggregation in the presence of SA. The zeta potential changes are shown in Figure 2D, where the 4-MPBA-modified AuNPs show a reduced zeta potential (−32.07 mV) compared with the colloidal AuNPs (−33.00 mV) due to the citrate ions that were displaced by 4-MPBA on the AuNPs’ surface. After further wrapping of the PEG_400_ on the AuNPs, a more negative charge can be observed in zeta potential (−34.57 mV). With the addition of different concentrations of SA, the zeta potential of the 4-MPBA-AuNPs@PEG_400_ system shows a positive shift owing to the anionic charges of SA (due to carboxyl groups) bound to the boronated anions. The UV–Vis spectra changes are shown in Figure 2E, where the surface plasmon band of the prepared 4-MPBA-AuNPs slightly red-shifted from 518 nm to 523 nm compared with initial AuNPs.

Meanwhile, a new broad peak was observed at around 660 nm, which shows the degree of aggregation of AuNPs. However, there is a 2 nm change for the absorption peak from 523 nm to 525 nm representing an aggregation, which is slightly reduced at 660 nm after the incubation of PEG_400_ on the AuNPs. Upon adding SA into the 4-MPBA-AuNPs@PEG_400_ solution, the peak intensity at 660 nm was largely increased immediately due to a quick increase in aggregation. DLS data also verified the above process, as seen in Figure S6; the mean size of crosslinking aggregation of 4-MPBA-AuNPs and 4-MPBA-AuNPs@PEG_400_ were increased compared with AuNPs. However, the average particle size of 4-MPBA-AuNPs@PEG_400_ increased significantly, indicating the aggregation states caused by the presence of SA. Figure 2F shows the Raman spectra of 4-MPBA-functionalized AuNPs. The typical SERS peaks assigned to 4-MPBA are found at 716 cm^−1^ (ν C-H), 1064 cm^−1^ (ν C-S, β C-C-C) and 1174 cm^−1^ (β B-OH). The bands at 1554 cm^−1^ (ν -OH) are ascribed to the original and O-H-associated forms of C-C stretching [36]. This behavior indicates that the AuNPs functionalized with the 4-MPBA group have been successfully prepared. Meanwhile, there is no obvious intensity change of the characteristic peak of 4-MPBA after incubation of PEG_400_ on the 4-MPBA-AuNPs’ surface, indicating that the active site for identifying analytes is retained.

### 3.3. Optimization of the Assay Conditions

The principle of the detection mechanism is the boronic acid from the 4-MPBA could selectively bind to the cis-diol groups to form a cyclic ester structure, which turns the solution from red to dark blue due to the aggregation of 4-MPBA-AuNPs.

#### 3.3.1. Effect of the Amount of 4-MPBA

**Figure 3:**
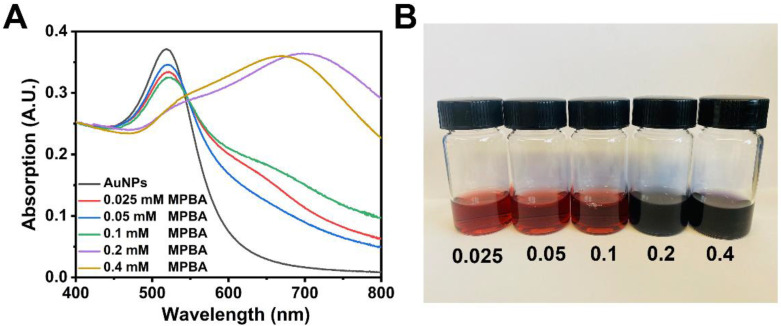
(**A**) UV–Vis absorbance responses of the AuNPs modified with increasing concentration from 0.025 to 0.4 mM 4-MPBA. (**B**) Photographs of their corresponding samples.

For the high-sensitivity detection of SA, the amounts of 4-MPBA and PEG_400_ of the SA colorimetric sensing system were considered. The 4-MPBA was modified on the AuNPs’ surface through the S-Au bond, and the amount of 4-MPBA was optimized to obtain the UV–Vis absorption peak. Firstly, various concentrations of 4-MPBA (from 0.025 mM to 0.4 mM) were used to modify the colloid AuNPs, as shown in Figure 3; when the concentrations of 4-MPBA increased from 0.025 to 0.1 mM, a slightly red shift peak can be observed at 523 nm, and the intensity of the absorption decreases with increasing concentrations. Once the concentration exceeds 0.1 mM, a new absorption peak caused by crosslinker aggregation can be found at 660 nm, which indicates that the 4-MPBA has a direct impact on the stability of the colloid AuNPs. The color of the 4-MPBA-AuNPs solution gradually changes from red to purple within this concentration range, as shown in Figure 3. Therefore, we selected 0.1 M 4-MPBA as the optimum concentration in the following studies.

#### 3.3.2. Effect of PEG on Sensing Performance

To regulate and control the interparticle crosslinking aggregation caused by 4-MPBA, a different molecular weight (MW)-type PEG was used. The MW 400 to 2000 PEG-modified on the 4-MPBA-AuNPs was used to detect various concentrations of SA, as shown in Figure S1. It is experimentally observed that the strength of the characteristic peak of 4-MPBA-AuNPs capture of SA at around 660 nm was obviously weakened with the increase of MW. Therefore, the smaller WM PEG is more conducive to retaining the ability of 4-MPBA -AuNPs to detect SA. Moreover, to obtain the best test performance of the 4-MPBA-AuNPs@PEG_400_, the amount of the PEG_400_ was also optimized. The PEG_400_ was negatively charged on the colloid AuNPs’ surface to control the 4-MPBA-AuNPs@PEG_400_, which was evenly dispersed and aggregated in the presence of SA. As shown from the data in Table S1, the sensitivity and detection range of the sensing system is better with the increase in the amount of PEG_400_. Furthermore, once the amount of PEG_400_ exceeds 0.05%, the test performance improvement is almost negligible, as shown in Figure S2, suggesting that 0.05% was regarded as the optimized amount of PEG_400_ for the assay.

#### 3.3.3. Optimization of Reaction Time

To evaluate the optimization of reaction time, the absorbance ratio (A_525_/A_660_) was examined after 2 mM SA was added to the 4-MPBA-AuNPs@PEG_400_ solution. As shown in Figure S3, the detection value was immediately observed in the presence of SA, and reached steady levels after 8 min. It is suggested that the as-mixed solution was maintained for 8 min for the following experiments.

### 3.4. Assay Performance of the 4-MPBA-AuNPs@PEG_400_ Sensing System for SA Detection

#### 3.4.1. The Linear Dynamic Range and Limits of Detection

**Figure 4:**
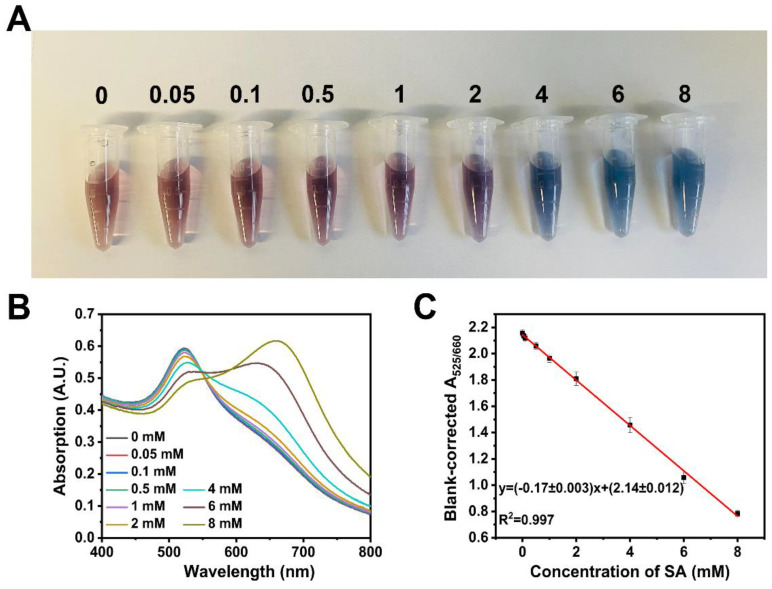
(**A**) Graph shows the color change by the different concentration of SA, (**B**) The UV–Vis absorption spectra of the 4-MPBA-AuNPs@PEG_400_ sensing system in the presence of various concentrations of SA. (**C**) The linear calibration plot for SA detections based on the changes in absorption (A_525/660_).

Under the optimal test conditions, we validated the sensitivity of the 4-MPBA-AuNPs@PEG_400_ sensing system for SA detection. As shown in Figure 4A, the color of the solution visible to the naked eye changes from red to blue as the concentration of SA increases. As shown in Figure 4B, a new absorbance peak was seen at approximately 660 nm, which indicates that 4-MPBA-AuNPs@PEG_400_ binds with SA, and the strength of the absorption peak is more obvious with the increase of the SA concentration, from 0.05 to 8 mM. The intensity of the A_525/660_ versus the SA concentration had a linear relationship, as shown in Figure 4C. The calibration equation can be described as *A*_525/600_ = −0.17 *C*_SA_ + 2.14, R^2^ = 0.997 (for the concentration range of 0.05–8 mM). The detection limit (LOD) of SA with this sensing system is estimated to be 48 μM.

Figure S4 compares the detection performance of the AuNPs and AuNPs@PEG_400_ with SA under the same experimental conditions, where we noticed a very weak response. As shown in Figure S5, in the 4-MPBA-AuNPs sensing system, the new peaks at 660 nm correspondingly increase with the increasing SA concentrations. The detection curve of SA is shown in Figure S5B, where the intensity of the A_523_/A_660_ is plotted as a function of the SA concentration. The results demonstrated that 4-MPBA specifically bound to SA is very useful in enhancing the sensitivity of SA detection. Nevertheless, compared to the 4-MPBA-AuNPs@PEG_400_ sensing system, the present sensor shows a relatively narrow dynamic linear range from 0.5 to 4 mM, which might be associated with the PEG_400_ improving detection performance. This mechanism can be explained as that the stability of stabilized AuNPs by DLVO is destroyed after modification with 4-MPBA and resulting in a crosslinking aggregation. In this case, 4-MPBA-AuNPs are easy to aggregate, even in the presence of a low concentration of SA.

Nevertheless, the repulsive force of the negative charge delayed the occurrence of aggregation after wrapping the negative charge PEG_400_ on the AuNPs’ surface, which provides more space to tolerate SA in the sensing system. Table 1 shows the comparison of the assay of the performance with the other SA sensor reported earlier; the 4-MPBA-AuNPs@PEG_400_ sensing system displays a lower limit of LOD and a wider linear range. More interestingly, such a detection range is enough to cover the physiological concentration of the healthy person (1.0–5.3 mM) [3], showing strong potential in the clinical application.

#### 3.4.2. Selectivity of the Sensing System

**Figure 5:**
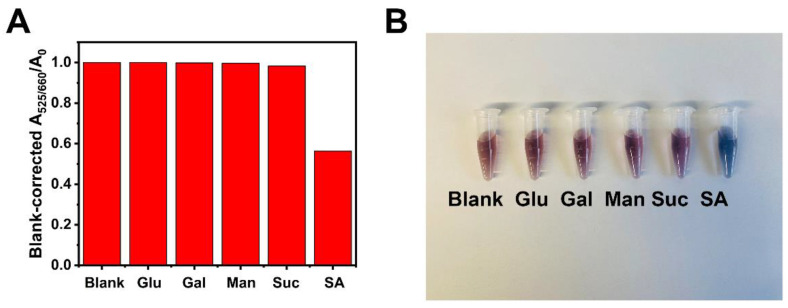
(**A**) Effects of different oligosaccharides, including the blank sample, 4 mM of glucose (Glu), galactose (Gal), mannose (Man), sucrose (Suc) and SA. (**B**) The graph shows the color change visible to the naked eye.

We assessed the selectivity of the present sensor by testing several oligosaccharides, including blank sample, glucose (Glu), galactose (Gal), mannose (Man) and sucrose (Suc) as control. As shown in Figure 5, the binding constant of SA on the 4-MPBA-AuNPs@PEG_400_ is significantly higher than other potential interfering sugars. There is an initial explanation for this: the trigonal form of the phenylboronic acid is more favorable for binding SA. At the same time, the tetrahedral boronated structure is better for binding other sugars. At a low pH (4.0 to 7.0), phenylboronic acid is in a trigonal structure; thus, the 4-MPBA-AuNPs@PEG_400_ had a high selectivity towards recognizing SA [15].

#### 3.4.3. Detection of Real Sample

The reliability of the proposed sensor for measuring SA was investigated in the rat serum samples and dried bird’s nests. No extraction steps other than the hydrolyzing polysialic acid were required before the assay of SA. The standard addition method analyzed by a calibration curve was adopted. Table 2 shows 94.4–100.1% and 92–112% recovery for bird’s nest, and serum samples were obtained. The results show that the 4-MPBA-AuNPs@PEG400 system-based colorimetric sensor can be used for the sensitive detection of SA in real biological and healthcare product samples.

## 4. Conclusions

In summary, we designed and synthesized novel AuNPs based on a colorimetric sensing platform by accurately controlling the balance between the electrostatic attraction and molecule interaction. The sensor was designed to contain two parts: the 4-MPBA was used for SA recognition, and the polymer PEG_400_ was used to stable AuNPs. The prepared 4-MPBA-AuNPs@PEG_400_ sensing platform was used for high selectivity and sensitivity detection of SA with a low detection limit of 48 μM. The present study provides a novel strategy to overcome the interparticle crosslinking aggregation probole of existing colorimetric sensors. Furthermore, the proposed sensor system exhibits excellent potential application in biological and healthcare product samples.

## Figures and Tables

**Table 1:** Performance comparison of different SA-sensing techniques.

Detection System	Liner Range (µM)	LOD (µM)	Analysis Time	Refs
4-MPBA-functionalized AuNPs	80–2000	68	10 min	[15]
3-APBA-functionalized AuNPs	80–250 or 300–700	35	10 min	[37]
3-APBA-AuNPs	150–1000	60	5 min	[38]
Chitosan-grafted polyaniline cryogel Electrochemical sensor	25–15,000	18	3 min	[39]
PDA liposome-based fluorescence turn-on sensor	400	14	-	[40]
4-MPBA-AuNPs@PEG_400_	50–8000	48	8 min	This work

**Table 2:** Determination of SA in bird’s nests and serum samples using the 4-MPBA-AuNPs@PEG_400_ sensing system.

Sample	Added (mM)	Found (mM)	RSD (%)	Recovery (%)
Bird’s nest	0	0.05	1.4	-
	0.1	0.151	3.3	101%
	0.5 1	0.503 0.992	4.6 2.3	99.6% 94.4%
Serum	0	3.00	0.86	-
	0.5	3.49	1.42	97.0%
	1	4.12	1.51	112.0%
	2	4.84	4.28	92.0%

## Data Availability

The data presented in this study are available within the article and its Supplementary Materials. Other data that support the findings of this study are available upon request from the corresponding author.

## Supplementary Materials

The following supporting information can be downloaded at: https://www.mdpi.com/article/10.3390/bios13020164/s1, Figure S1: The sensing performance of the 4-MPBA-AuNPs with modified with different molecular weight (MW)-type PEGs; Figure S2: Different concentrations of PEG_400_ coated on 4-MPBA-AuNPs for the detection of SA.; Figure S3: Optimization of reaction time; Figure S4: AuNPs and 4-MPBA-AuNPs system for the detection of SA; Figure S5: (A) Extinction spectra of 4-MPBA-AuNPs at various SA concentrations, (B) Plot of A_523/660_ against SA concentration; Figure S6: The DLS analysis of the AuNPs, 4-MPBA-AuNPs, 4-MPBA-AuNPs@PEG_400_ and 4-MPBA-AuNPs@PEG_400_ mixed with SA; Table S1: The sensitivity of the 4-MPBA-AuNPs coated with various concentration PEG_400_ sensing platform for the detection of SA title.
