# Supplementary material for: A Schematic Colorimetric Assay for Sialic Acid Assay Based on PEG-Mediated Interparticle Crosslinking Aggregation of Gold Nanoparticles
Source: Biosensors (Basel). 2023 Jan 20;13(2):164. doi: 10.3390/bios13020164 (PMC9953623; doi:10.3390/bios13020164)
Supplement: Supplementary file 1 [file biosensors-13-00164-s001.zip › biosensors-2144061-supplementary.pdf]

biosensors

MDPI

Supplementary Materials

A schematic colorimetric assay for sialic acid assay based on PEG-mediated interparticle crosslinking aggregation of gold nanoparticles

Shixing Tang [1,2,3,*] , Lin Li [1,2], Rui Wang [1,2], Sagar Regmi [4], Xinyu Zhang [2], Guoqiang Yang [1,2,5,*], and Jian Ju[2,6,*]

[1] School of Ophthalmology & Optometry, Wenzhou Medical University, Wenzhou 325035, China

[2] Engineering Research Center of Clinical Functional Materials and Diagnosis & Treatment Devices of Zhejiang Province, Wenzhou Institute, University of Chinese Academy of Sciences, Wenzhou 325001, China

[3] Department of Epidemiology, School of Public Health, Southern Medical University, Guangzhou 510515, China

[4] Department of Pharmacology, School of Medicine, Case Western Reserve University, Cleveland, OH 44106, USA

[5] Key Laboratory of Photochemistry, Institute of Chemistry, University of Chinese Academy of Sciences, Chinese Academy of Sciences, Beijing 100190, China

[6] Oujiang Lab, Wenzhou 325001, China

*Correspondence: tamgshixing@smu.edu.cn (S.T.); gqyang@iccas.ac.cn (G.Y.); jujan@ucas.ac.cn (J.J.)

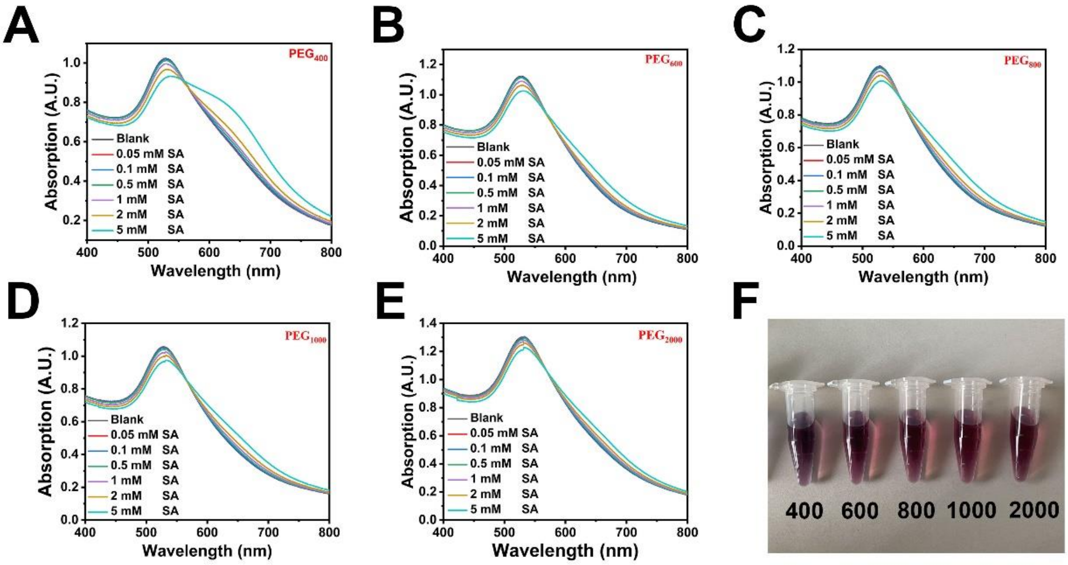

Figure S1. The sensing performance of the 4-MPBA-AuNPs with modified with different molecular weight (MW) type PEG. (MW: from 400 to 2000). The graph of the different type 4-MPBA-AuNPs-PEG solutions.

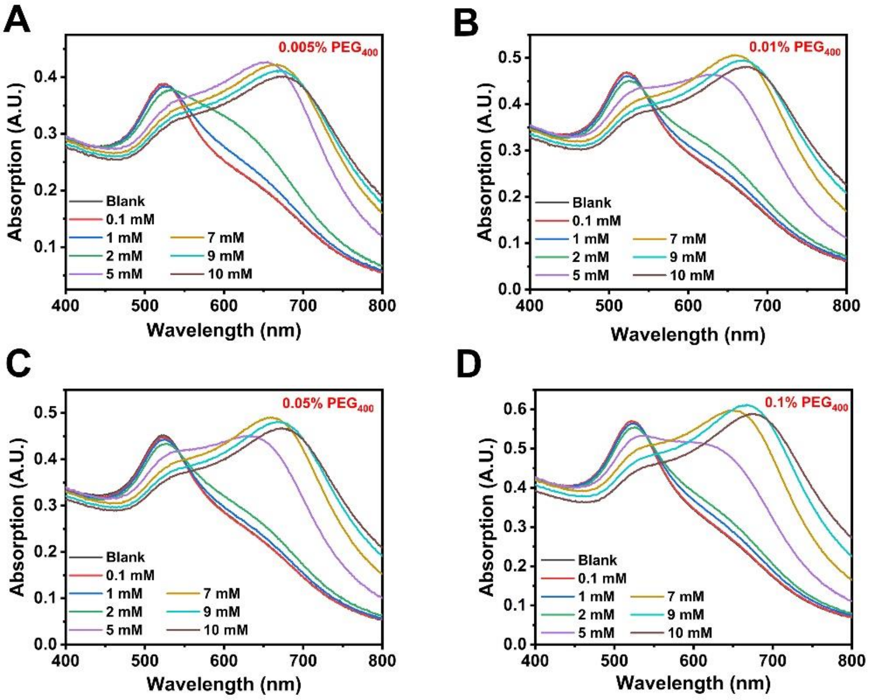

Figure S2. The UV-Vis spectrum of different concentrations of PEG_{400} coated on 4-MPBA-AuNPs for the detection of SA.

Table S1. The sensitivity of the 4-MPBA-AuNPs coated with various concentration PEG_{400} sensing platform for the detection of SA. ($A_{525/660}$ vs Concentration).

	0.005%	0.01%	0.05%	0.1%
Slope	*-0.196*	-0.206	-0.214	-0.216
R^2	*0.944*	0.984	0.98	0.997

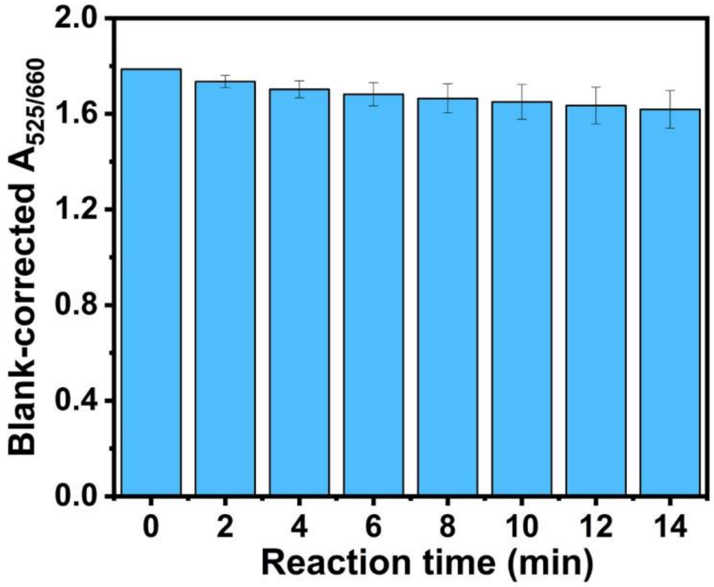

Figure S3. The ratio of the absorbance of 2 mM SA incubated with 4-MPBA-AuNPs@PEG_{400} from 0 to 14 min.

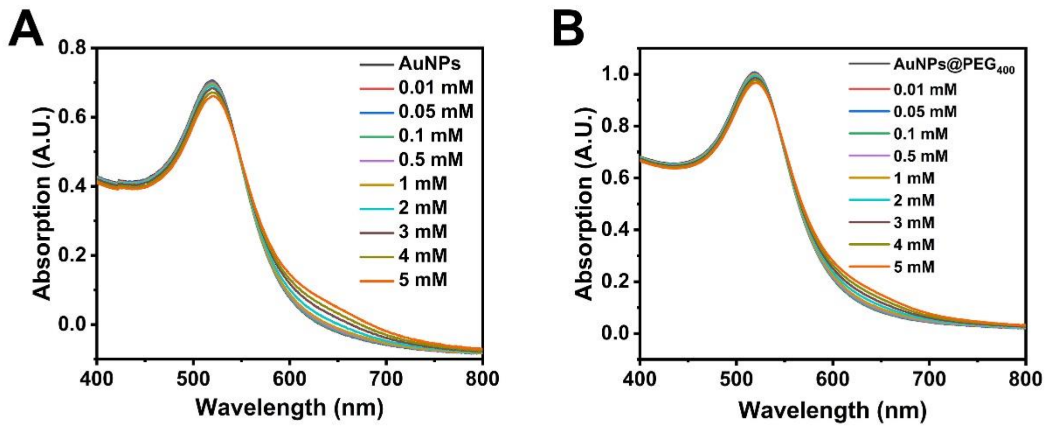

Figure. S4. (a) Extinction spectra of AuNPs at various SA concentrations, (b)extinction spectra of AuNPs@PEG_{400} at various SA concentrations.

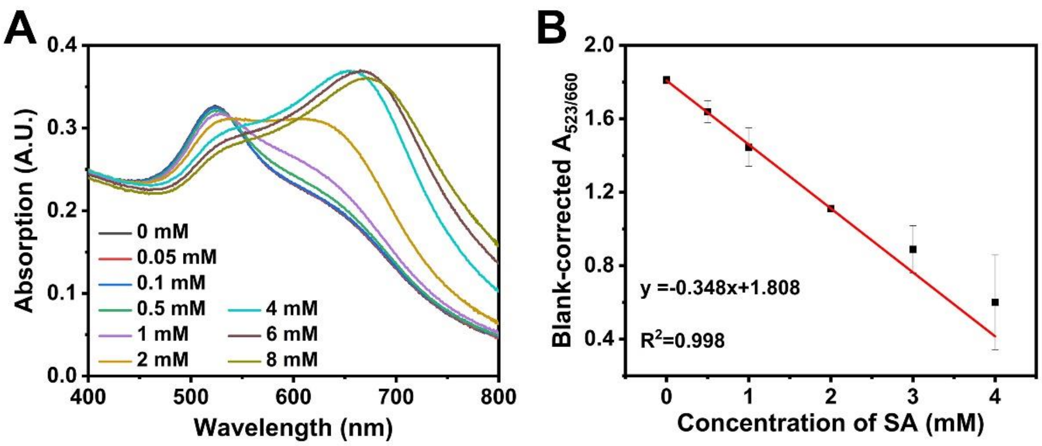

Figure. S5. (A) Extinction spectra of 4-MPBA-AuNPs at various SA concentrations, (B) Plot of $A_{523/660}$ against SA concentration.

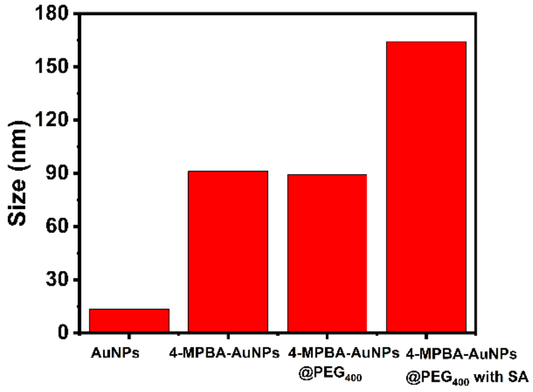

Figure. S6. The DLS analysis of the AuNPs, 4-MPBA-AuNPs, 4-MPBA-AuNPs@PEG_{400} and 4-MPBA-AuNPs@PEG_{400} mixed with SA.
